# Effects of Educational Intervention on Dental Plaque Index in 9-Year-Old Children

**DOI:** 10.1155/2022/7339243

**Published:** 2022-10-25

**Authors:** Mahdieh Zarabadipour, Mehrnaz Makhlooghi Sari, Alireza Moghadam, Benyamin Kazemi, Monirsadat Mirzadeh

**Affiliations:** ^1^Department of Oral and Maxillofacial Medicine, Dental Caries Prevention Research Center, Qazvin University of Medical Sciences, Qazvin, Iran; ^2^Student Research Committee, Qazvin University of Medical Sciences, Qazvin, Iran; ^3^Department of Community Medicine, Metabolic Diseases Research Center, Research Institute for Prevention of Non-Communicable Diseases, Qazvin University of Medical Sciences, Qazvin, Iran

## Abstract

**Methods:**

119 students of third-grade elementary (65 girls and 54 boys) from government and private schools of Qazvin city participated. The dental plaque index of every participant was primarily recorded by Silness and Loe method. Students were trained by a dental student by face-to-face method, and after 2 weeks, dental plaque indices were recorded again for every individual. After index recording and coding, data analysis was done using SPSS version 21.

**Results:**

Results showed that the dental plaque index was statistically significantly different before and after training (*p* < 0.001).

**Conclusion:**

Based on the results of this study, students' motivation in addition to proper training, can have a significant impact on oral and dental health. Furthermore, this study shows that face-to-face training along with involving the individual in training and learning was seen as quite helpful.

## 1. Introduction

Oral health in each person depends on several factors; the use of mechanical instruments to effectively maintain oral hygiene can play a role in advancing this goal [1], such as manual and electric toothbrushes, better improvement in clinical parameters has been observed in sonic action heads toothbrushes as a type of electric [2]. In addition, knowledge of preventive methods and periodic dental recalls complement this importance. To provide effective and efficient oral health training, it is recommended to start health education at an early age [3, 4]. This training, if done properly and correctly, will become an effective habit and will eventually manifest itself in a healthy and disease-free mouth [5, 6].

A variety of methods vis-a-vis health education have been mentioned in different studies [7, 8]. For example, Barak et al.'s study report that picture-based education plays an important role in children's health learning [9]. Certain articles have stated that if the learner is involved in the training, it is much more effective than if the training is based solely on a model or video [10–12]. Also, Basir et al. have reported the effect of health education on the health status of periodontium [13].

Poor oral hygiene is a known etiological factor for oral diseases including dental caries and periodontal disease [8]. The simplest way to assess oral hygiene is to evaluate periodontal health by examining the amount of dental plaque formed [14]. Dental plaque is a bacterial layer that adheres to tooth surfaces, so the most effective method for its removal is mechanical hygienic methods [15]. For this reason, toothbrushes and floss are the most important tools for oral health and their use can be the simplest self-care behavior in oral health [16].

The aim of this study was to evaluate the dental plaque index before and after educating primary school children about oral health and hygiene.

## 2. Materials and Methods

To conduct this study, a list of primary schools in urban areas of Qazvin city, Iran, was prepared using a multistage cluster sampling method, and thereby, five primary schools were randomly selected (3 public schools and 2 private schools). And in each primary school, 30 students from the third grade of elementary school (approximately 9 years old) were randomly selected based on an alphabetically arranged list. Criteria for students' inclusion in the study were appropriate general and mental health and parental consent for their ward's participation in the study. Exclusion criteria consisted of students who were on an antibiotic therapy or any oral medication during the span of the study.

On the first day of their referral, the dental plaque index was determined (as a basis, PI-1) for each person by Silness and Loe method. The measurement of this index is based on the recording of both soft and mineral accumulations on the teeth. Each of the four tooth surfaces i.e., mesiobuccal, midbuccal, distobuccal, and midlingual secured a score of 0, 1, 2, and 3, respectively. Afterward, this score was added together and averaged. Below mentioned are the grades assigned and their interpretation.

  Grade 0: no plaque.
  Grade 1: accumulation of plaque in the free margin of the gums and lateral parts of the tooth (existing plaque may only be detected with the help of a probe).
  Grade 2: moderate accumulation of soft deposits in the gingival pocket or on the teeth and gingival margin, which can be seen with the naked eye.
  Grade 3: frequency of soft accumulations in the gingival envelope or on the teeth and gingival margin.

Health education was conducted by a trained dental student in a face-to-face training method with the cooperation of students. Thus, 16 steps were set for brushing the mouth. These 16 steps were meant to include internal, external, and masticatory surfaces of all anterior and posterior teeth in both jaws. Then, the same toothbrushes and same toothpaste were distributed equally among all the students in the form of health packs and they were asked to brush in the same way on the same day and in the same place. The oral hygiene method was controlled individually. Students were asked to brush twice a day for 4 minutes without parental help and in the manner that was taught to them, and finally, after two weeks, the dental plaque index was recorded for each participant (PI-2).

After collecting and encoding the data, the data were analyzed by SPSS version 21. The error of the first type (a) was considered to be 0.05 and p < a was considered significant.

## 3. Results and Discussion

### 3.1. Results

This study involved 119 children aged 9 years old from different urban areas of Qazvin city, chosen from an initial number of 150 randomly selected individuals. Additionally, 31 people were excluded from the study for reasons such as lack of cooperation, the presence of a systemic disease, and parental dissatisfaction. Of these, 65 were girls and 54 were boys.  Table 1 shows the sex distribution of students participating in the study.

Information related to dental plaque index before and after the training intervention and its comparison by *t*-test has been expressed in Table 2. The average of registered primary dental plaque (PI-1) was 1.161 (with a standard deviation of 0.594), which became 0.784 (with a standard deviation of 0.505) after the intervention.

Data analysis for plaque index showed a statistically significant (*p* < 0.001) difference before and after the intervention.

## 4. Discussion

Oral health has a clear impact on the health of the body. The health of oral tissues depends on the effective removal of debris and plaque from dental surfaces. Observing this important issue in children is possible with continuous education and adherence. Awareness of the child about the importance and necessity of oral hygiene along with effective health education can play an important role in improving dental and oral tissue health [14].

The current study was performed in 2019 and involved 119 primary school students from different areas of Qazvin. According to the results of the present study, the dental plaque index of students before the training intervention was at a moderate level. Although, with face-to-face training and students' cooperation, this amount was significantly reduced and dental plaque index before and after the study had a statistically significant difference.

The positive effect of one-on-one health education has been confirmed in other studies, despite being time-consuming and energy-intensive. Ahmad et al.'s study state that the inclusion of health education in children's educational curriculum, which includes brushing techniques, can have a significant impact on children's oral health [5]. Raj et al.'s study also found that after an effective health education, the percentage of students who brushed regularly and daily increased from 4.1% to 9.9%. In his study, the number of students who never brushed their teeth significantly decreased after the educational intervention [17]. These results are similar to the results of Hajimiri et al. [3] and Ferrazzano et al. [16].

The health educational method used in this study was face-to-face and practical education with the participation of students. Based on the literature, the most basic method of health education is the method in which the learner is involved in education. The Alhayek et al. study compared two traditional teaching methods with animation-based teaching to improve children's oral health. In their study, it was stated that the use of animation in educating children was associated with less effort in education and was more understandable and tangible for children [10].

Angelopoulou et al. study employed a similar method, comparing traditional methods of health education with experimental methods combined with a seminar training teachers and learners' involvement in education. The study also confirms the effect of new education in improving students' oral health [11]. However, Ahmad et al. study used the lecture method, which is the simplest, most accessible, and most common method of health education [5].

The use of face-to-face teaching methods is strongly dependent on the skill of the instructor. In Hendi et al.'s study, it is stated that if the instructor-student is taught in standard methods, learners can be brought to the desired practical skills [15]. Khorakian et al. have also reported the effect of using a trained student more than the teacher, which can be due to the closest match between the age of the educator student and the learners, which increases the effect of words and doubles the motivation to learn [12]. In Haleem et al.'s study, the effectiveness of health education by a trained student was equivalent to that provided by a dentist and a teacher, and perhaps greater acceptance of this type of education was recorded by learners [18].

Eley et al. study found that students' knowledge of proper hygiene was poor, but proper training by a trained and experienced person could play an important role in improving oral health [7]. In Angelopoulos et al. study, 9-year-old students were asked to participate. According to the research conducted thus far, students of this age have the ability to be taught, have the ability of logical thinking, and can participate in group work and analyze the results [11]. Jain et al. have also examined the health education of teachers in the same age group [4].

One of the limitations of the present study was the time limit and access to students to re-evaluate the impact of this training on oral health survival because it coincides with the Covid-19 pandemic. Also, the decrease in the number of samples from 150 to 119 was due to the exclusion of students from the study due to a variety of reasons such as illness and drug use, lack of cooperation in observing the principles of the study, and absence in the second examination session. Due to the pandemic, it was not possible to continue the study and resampling. Also, the release of the results was delayed in the hope of ending the pandemic.

Given the potential for temporary effects of health education, which has been confirmed in other studies [13], we suggest that frequent and accessible review training be provided to students and families. Also, a systematic review study conducted by Stein has shown that the long-term effects of this education on children are not very obvious and there is a need for repeated studies in this field [1]. On the other hand, strict observance of hygienic points and standard protocol will be more effective with parental supervision and education, and it is recommended that in future studies, parents' participation and support in carrying out the study should be motivated. In the next studies, in addition to using mechanical methods and face-to-face training for dental plaque control, it is suggested to use remineralizing agents, such as biomimetic zinc-substituted hydroxyapatite for the remineralization of the enamel surface and reduce the incidence of dental caries [19].

## 5. Conclusions

Based on the present study, encouraging and creating positive motivation among students along with proper teaching of oral hygiene methods can play a significant role in oral and dental health. This study also states that using face-to-face training methods along with involving the individual in learning is very helpful.

## Figures and Tables

**Table 1:** Gender distribution of participants.

	Number	Percent
Male	54	45.4
Female	65	54.6
Total	119	100

**Table 2:** Comparison of the dental plaque index before and after the educational intervention.

Plaque index	Mean	Standard deviation	*p* value
PI-1	1.161	0.594	<0.001
PI-2	0.784	0.505	

## Data Availability

The data used to support the findings of this study are included within the article.
